# Pipette tip with integrated electrodes for gene electrotransfer of cells in suspension: a feasibility study in CHO cells

**DOI:** 10.2478/v10019-011-0025-4

**Published:** 2011-08-26

**Authors:** Matej Rebersek, Masa Kanduser, Damijan Miklavcic

**Affiliations:** University of Ljubljana, Faculty of Electrical Engineering, Ljubljana, Slovenia

**Keywords:** electrodes, gene electrotransfer, pipette tip, CHO cell line

## Abstract

**Background:**

Gene electrotransfer is a non-viral gene delivery method that requires successful electroporation for DNA delivery into the cells. Changing the direction of the electric field during the pulse application improves the efficacy of gene delivery. In our study, we tested a pipette tip with integrated electrodes that enables changing the direction of the electric field for electroporation of cell suspension for gene electrotransfer.

**Materials and methods:**

A new pipette tip consists of four cylindrical rod electrodes that allow the application of electric pulses in different electric field directions. The experiments were performed on cell suspension of CHO cells in phosphate buffer. Plasmid DNA encoding for green fluorescent protein (GFP) was used and the efficiency of gene electrotransfer was determined by counting cells expressing GFP 24 h after the experiment.

**Results:**

Experimental results showed that the percentage of cells expressing GFP increased when the electric field orientation was changed during the application. The GFP expression was almost two times higher when the pulses were applied in orthogonal directions in comparison with single direction, while cell viability was not significantly affected.

**Conclusions:**

We can conclude that results obtained with the described pipette tip are comparable to previously published results on gene electrotransfer using similar electrode geometry and electric pulse parameters. The tested pipette tip, however, allows work with small volumes/samples and requires less cell manipulation.

## Introduction

Gene electrotransfer is a non-viral method used to transfer DNA molecules into living cells by means of high-voltage electric pulses.^1^–^6^ It is proven to be effective *in vitro*^7^ and it has great potential for *ex vivo* transfection.^8^ In comparison to viral methods it is safer^9^ but less efficient *in vivo.*^10^,^11^ However, being extensively investigated, gene electrotransfer is becoming a promising non-viral gene therapy method.^12^–^17^ For *in vitro* and *ex vivo* experiments higher transfection yield can be achieved by optimizing electroporation medium, DNA preparation and its concentration, and parameters of electric pulses. The transfection yield obtained by gene electrotransfer *ex vivo* for hematopoietic and stem cells is still a problem, therefore, further optimisation of the protocol is needed.^18^,^19^ One of the possible optimization options lies in increasing the area of cell membrane that is competent for the uptake of the plasmid DNA.

Study of Golzio *et al.* in 2002 demonstrated for the first time that during electric pulse application complex between DNA and permeabilized cell membrane was formed. The complex is formed only on the side of the cell facing anode indicating that DNA molecule only enters the cell on the membrane facing anode, therefore, changing the direction of the electric field results in the increase of the membrane area that is competent for DNA entry into the cell.^20^ Afterwards, it was demonstrated that changing the electric field direction during electric pulse delivery improved the efficiency of gene electrotransfer *in vitro*.^21^–^24^

The aim of our research was to improve the design of electrodes that allow the application of electric pulses in different directions for electroporation of cell suspensions. We designed and tested a new pipette tip with integrated electrodes that could be used for improved gene electrotransfer *in vitro* and *ex vivo* for hematopoietic and stem cells.

## Materials and methods

### Cell cultures

Chinese hamster ovary CHO cells (European Collection of Cell Cultures) were grown in a nutrient mixture Ham’s F12 (PAA, Austria) supplemented with 2 mM L-glutamine (Sigma-Aldrich, Germany), 10% foetal bovine serum (Sigma-Aldrich, Germany) and antibiotics Penicillin/ Streptomycin and Gentamicin (PAA, Austria). Cells were kept at 37°C in a humidified 5% CO_2_ atmosphere in the incubator for 3 to 4 days. Cell suspension was prepared by trypsinization of 90% confluent cell culture that was centrifuged for 5 minutes at 4°C. Cell pellet was resuspended in iso-osmolar phosphate buffer with pH 7.4 consisting of 10 mM Na_2_HPO_4_/NaH_2_PO_4_, 1 mM MgCl_2_ and 250 mM sucrose.

### Gene electrotransfer protocol

**FIGURE 1:**
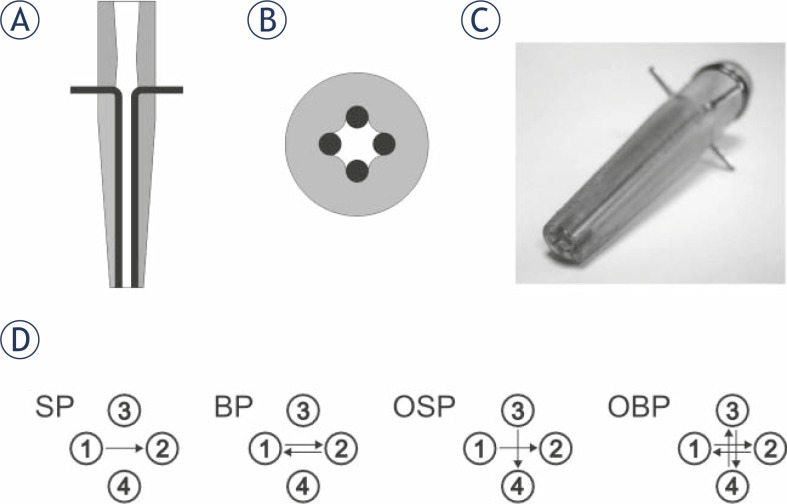
Pipette tip and electric field protocols. Vertical (A) and horizontal (B) cross section and photograph (C) of pipette tip with integrated electrodes. In the cross section grey colour is used for the plastic housing and black for the electrodes. Electric field protocols (D): in single polarity (SP) 8 electric pulses were applied in one direction between electrodes 1 and 2 (8 pulses in one direction), in both polarities (BP) 8 electric pulses were applied in both directions between electrodes 1 and 2 (4 pulses in each direction), in orthogonal single polarity (OSP) 8 electric pulses were applied in one direction between electrodes 1 and 2, and 3 and 4 (4 pulses in each direction), and in orthogonal both polarities (OBP) 8 electric pulses were applied in both directions between electrodes 1 and 2, and 3 and 4 (2 pulses in each direction).

Cells were exposed to the electric field in the pipette tip with integrated electrodes connected to a high-voltage prototype generator. The pipette tip with integrated electrodes for electroporation of cell suspension consists of four cylindrical rod electrodes and allows the application of relatively homogeneous electric field in different directions (Figure 1A,B,C). The electrodes are made of 90% platinum / 10% iridium; their diameter is 1.4 mm, adjacent electrodes are 1 mm apart, and opposite electrodes are 2 mm apart. The electrodes are glued into the plastic tip in parallel and their applicable length is 30 mm, so that the maximal treatable volume of cell suspension could be 140 μl. Numerical calculations of electric field distribution for four cylindrical rod electrodes were presented in our previous publication^22^ and could be scaled down to smaller geometry in the presented pipette tip. The tip and the generator were developed at Laboratory of Biocybernetics, Faculty of Electrical Engineering, University of Ljubljana described in detail in patent^25^ and previous publication.^22^

In our experiment, four different electric field protocols were used (Figure 1D): single polarity (SP), both polarities (BP), orthogonal single polarity (OSP) and orthogonal both polarities (OBP). In each electric field protocol 8 electric pulses were used in total. When SP electric field protocol was used, 8 pulses were applied in one direction between two opposite electrodes only. When BP electric field protocol was used, both polarities electric pulses were applied between two electrodes, 4 pulses in each direction. When OSP electric field protocol was used, single polarity electric pulses were applied between two orthogonal pairs of electrodes, 4 pulses in each direction. And when OBP electric field protocol was used, both polarities electric pulses were applied between two orthogonal pairs of electrodes, 2 pulses in each direction. Besides cells treated with different electric pulse protocols, cells not treated with electric pulses were used as control.

The pipette tip was sterilised before experiments in 70% ethanol for 10 minutes and rinsed thoroughly in sterile pulsing buffer before the first sample was treated and then the tip was rinsed each time before new electric field protocol was applied. For each parameter 100 μl of cell suspension was aspirated into the tip. In preliminary experiments electric pulse amplitude in the range from 80 to 300 V were tested and for further experiments 200 and 225 V were selected. Treated cells were exposed to 8 electric pulses with amplitude 200 V or 225 V, distance between the applied electrodes was 2 mm, pulse duration 1 ms and repetition frequency of 1 Hz regardless of the electric field protocol that was used *i.e.* 8 pulses in one direction for SP, 4 pulses in each direction for BP and OSP, and 2 pulses in each direction for OBP.

The efficiency of gene delivery was measured with the expression of reporter gene green fluorescent protein (GFP) 24 hours after gene delivery. Plasmid DNA pEGFP-N1 encoding for GFP was prepared with Endofree Plasmid mega kit (Qiagen, USA). The plasmid DNA was added to cell suspension that contained 5 × 10^5^ cells/ml so that the final plasmid concentration was 10 μg/ ml.^26^ The mixture was then incubated for 2–3 minutes at room temperature. For electroporation 100 μl of cell suspension was aspired into the pipette tip and different electric pulse protocols were applied. After the pulse application treated cells were incubated for 5 minutes at 37°C to allow cell membrane resealing. Cells were then plated in a nutrient mixture Ham’s F12 supplemented with 2 mM L-glutamine, 10% foetal bovine serum and then grown for 24 hours in cell culture medium to allow GFP expression.

Efficiency of transfection was determined as the percentage of cells expressing GFP. Cells were observed by fluorescent microscopy (Zeiss 200, Axiovert, ZR Germany). Excitation light for GFP detection was 488 nm (monochromator system PolyChrome IV, Visitron, Germany) and emission was 508 nm (Chroma, Rockingham, USA). Besides fluorescent images for each experimental condition phase contrast images were acquired by MetaMorph imaging system (Visitron, Germany) at 20x objective magnification. At least 5 randomly chosen images were recorded and analysed per each experimental condition. The cells were counted manually and the transfection efficiency was determined by the ratio between the number of fluorescent cells that express GFP and the total number of cells present in a phase contrast image. The approximate number of cells counted per each image was 300. The short term cell survival was determined 24 hours after the electric pulse treatment as the ratio between the number of cells present in the control and the number of cells counted in the treated sample exposed to electric pulses. For each experimental protocol at least three independent experiments were performed. Results are presented as mean values ± standard deviation.

### Statistics

Statistical tests One way analysis of variance (One Way ANOVA) were performed on all results (SigmaStat 3.1, Systat, USA). Bonferroni t-test was performed on results if there was indication of a statistically significant difference between different electric field protocols used.

## Results and discussion

**FIGURE 2:**
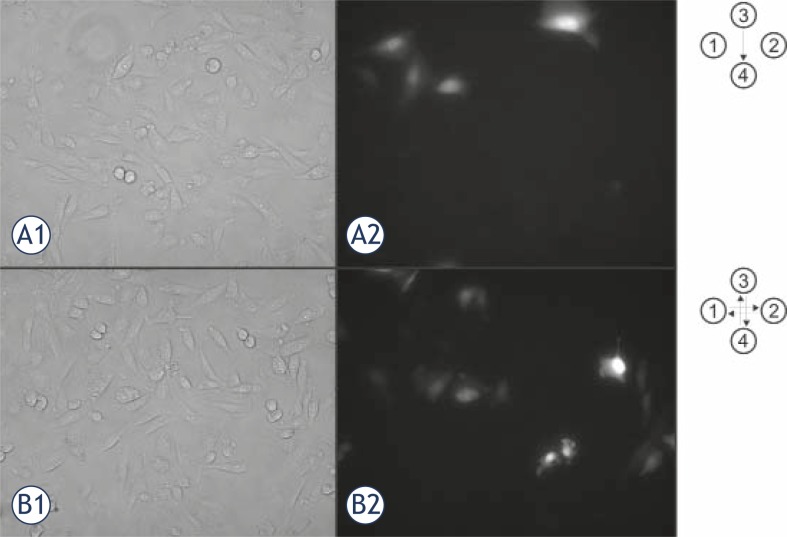
Phase contrast (A1 and B1) and fluorescence images (A2 and B2) of CHO cells 24 h after exposure to electric pulses at 20x objective magnification. Cells were exposed to 8 rectangular pulses with amplitude 225 V. The distance between the applied electrodes was 2 mm. Two electric field protocols are presented: single polarity (SP, A1 in A2), and orthogonal both polarities (OBP, B1 and B2). Only efficiently transfected cell express green florescence due to expression of GFP plasmid that was introduced into the cell by electric pulses (A2 and B2).

**FIGURE 3:**
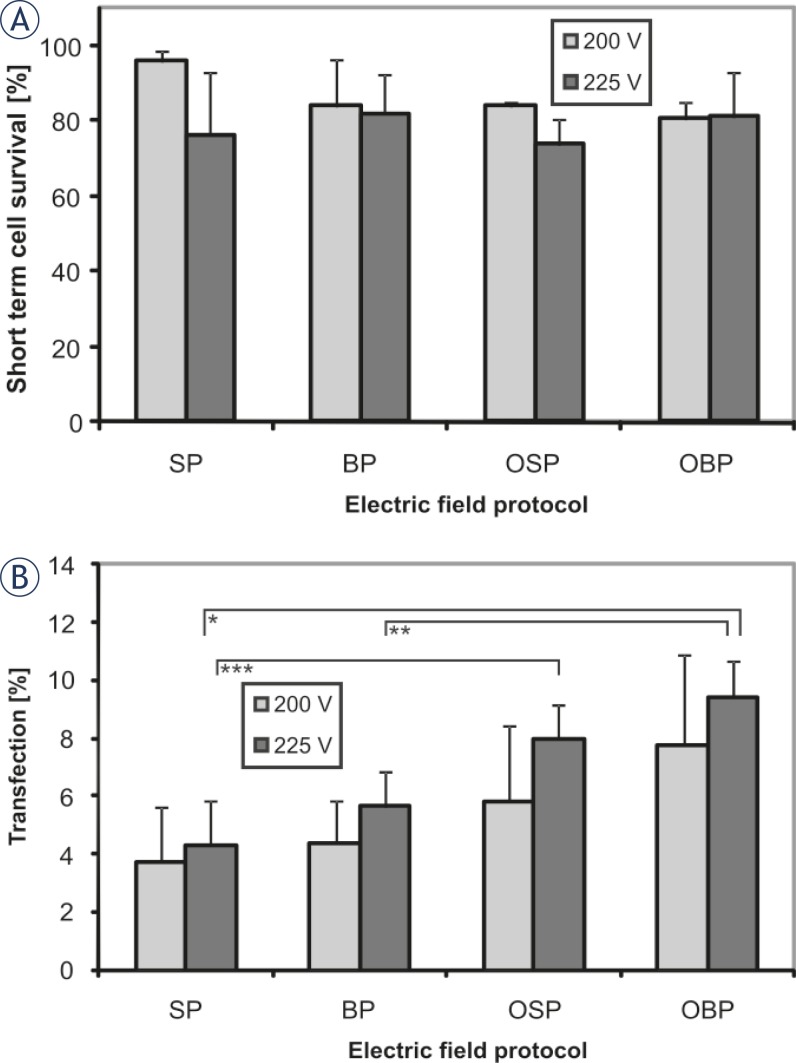
Influence of different electric field protocols on short term cell survival and transfection efficiency 24h after exposure to electric pulses. The cell survival (A) and the percentage of cells expressing GFP (B) for single polarity (SP), both polarities (BP), orthogonal single polarity (OSP) and orthogonal both polarities (OBP) electric field protocol. Cells were exposed to a train of eight pulses with amplitude 200 V and 225 V, duration 1 ms and repetition frequency of 1 Hz. Results were obtained by means of fluorescence microscopy. Each value in the graph represents mean of three independent experiments ± standard deviation. Statistical significance is marked by * (P = 0.008), ** (P = 0.042), and *** (P = 0.05). Changing the electric field orientation during the pulse application has increased the percentage of cells expressing GFP (B) whereas the cell viability remained the same (A).

The aim of our research was to design and test the new pipette tip with integrated electrodes and to evaluate the efficacy of the pipette tip for gene electrotransfer for 4 different electric field protocols. Experimental results are presented in Figures 2 and 3. In Figure 2 phase contrast and fluorescence micrographs of CHO cells expressing GFP 24 hours after the electric pulse application are presented. The upper row represents cells treated with electric pulses of single polarity (SP) while the bottom row shows cells treated with pulses applied in orthogonal positions of electrodes and in both pulse polarities (OSP). Figure 3 shows cell viability and percentage of cells expressing GFP observed 24 hours after the treatment of cell suspension with electric pulses in the pipette tip with integrated electrodes. The cells were treated with 8 electric pulses regardless of the electric field protocol that was used. We have presented the results for 200 V and 225 V (d = 2 mm) as with these voltages the transfection yield was the highest. With lower voltages the percentage of transfected cells was lower and with higher voltages cell viability decreased while the differences between pulse protocols remained (data not shown). In Figure 3A it can be seen that the cell viability was not significantly affected by changing the electric field direction during the application of electric pulses. For cell viability statistical test One way ANOVA indicated that there was no statistically significant difference between the electric field protocols (P = 0.093 for 200 V and P = 0.798 for 225 V, d = 2 mm). The percentage of cells expressing GFP increased when the electric field direction was changed during the application of electric pulses (Figure 3B). Average percentage of cells expressing GFP was approximately two times higher when the pulses were applied in orthogonal directions of both polarities (OBP) in comparison with single direction and polarity (SP). For gene electrotransfer One Way ANOVA indicated that there was a statistically significant difference between the electric field protocols for 225 V (P = 0.005) but not for 200 V (P = 0,237). For 225 V Bonferroni t-test indicated that there was a statistically significant difference when we compared orthogonal both polarities (OBP) versus single polarity (SP) electric field protocol (P = 0.008) and orthogonal both polarities (OBP) versus both polarities (BP) electric field protocol (P = 0.042). Also the comparison of orthogonal single polarity (OSP) versus single polarity (SP) electric field protocol gave statistically significant difference (P = 0.05) while for other combinations the difference was not statistically different (P > 0.05).

Experimental results obtained with the pipette tip with integrated electrodes for electroporation of cell suspension show that the percentage of cells expressing GFP increases when the electric field direction was changed during the application of electric pulses whereas the cell viability was not affected. The results are in accordance with previously published results using similar electrode geometry and electric pulse parameters^21^,^22^ indicating that the new pipette tip can be successfully used for gene electrotransfer. The advantage of this new pipette tip is easier and quicker cell handling during the electroporation experiment, and better control of the volume of cell suspension exposed to electric field in comparison with electrode design described in our previous paper.^22^ Previous design was suitable for the electroporation of plated cells and presented some drawbacks when used for cell suspensions. Cell suspension was applied as a droplet among 4 rod electrodes and the main drawback was that the shape of the drop was not as uniform as the shape of the liquid in the tips presented here. The consequence of the uneven droplet shape was that not all the cells in the droplet were exposed to the homogeneous electric field. Therefore, the percentage of transfected cells was not consistent. This drawback was overcome by incorporation of the electrodes into the walls of the pipette tip. In addition the tip allows working with small volume of cell suspension which may be particularly important in treating of valuable cells or plasmid. However, the aim of our work was to demonstrate proof of principle. Therefore, in our experiments cell suspension volumes of 100 μl were used to compare our data with the data used earlier. Nevertheless, the current tips allow working with smaller volumes down to 30 μl and the tips design could be scaled down to even smaller sizes.

In conclusion, the new pipette tip with integrated electrodes can be successfully used for gene electrotransfer, as obtained results are comparable with previously published results. The advantage of this new pipette with integrated electrodes is that it allows handling of small volumes/samples and requires less cell manipulation in comparison to established methods.
